# Myositis ossificans traumatica of the masticatory muscles: etiology, diagnosis and treatment

**DOI:** 10.1186/s13005-018-0180-6

**Published:** 2018-10-29

**Authors:** Marcel Hanisch, Lale Hanisch, Leopold F. Fröhlich, Richard Werkmeister, Lauren Bohner, Johannes Kleinheinz

**Affiliations:** 1 Department of Cranio-Maxillofacial Surgery, Research Unit Rare Diseases with Orofacial Manifestations (RDOM), University Hospital Münster, Albert-Schweitzer-Campus 1, Gebäude W 30, D-48149 Münster, Germany; 2 Department of Orthodontics, Faculty of Health, School of Dentistry, Witten/Herdecke University, Alfred-Herrhausen-Strasse 44, 58455 Witten, Germany; 3 Department of Cranio-Maxillofacial Surgery, AG VABOS, University Hospital Münster, Albert-Schweitzer-Campus 1, Gebäude W 30, D-48149 Münster, Germany; 4 Department of Oral and Maxillofacial Surgery, Central German Armed Forces Hospital, Rübenacher Strasse 170, 56072 Koblenz, Germany

**Keywords:** Myositis ossificans, Myositis ossificans traumatica, Myositis ossificans circumscripta, Heterotropic ossification, Masticatory muscles

## Abstract

**Background:**

Myositis ossificans describes a heterotopic bone formation within a muscle. Thereby myositis ossificans is classified in two different groups: myositis ossificans progressiva (MOP) which describes a genetic autosomal dominant rare disease and myositis ossificans traumatica (MOT). The exact pathogenesis of MOT is unclear. The aim of this article was to analyse and interpret the existing literature reporting MOT of masticatory muscles and compare the results with our own clinical experience with MOT. Risk-factors, etiology, clinical features, diagnostic imaging, as well as different treatment options were evaluated and recommendations for the prevention, diagnosis, and therapy of MOT of the masticatory muscles were given.

**Methods:**

Following the PRISMA-Guidelines, a systematic search within the PubMed/Medline database with a view to record literature of MOT of the masticatory muscles was performed. Furthermore, the database of our own clinic was screened for cases of MOT.

**Results:**

In total, 63 cases of MOT of the masticatory muscles which were reported in English-based literature were included in this study. Overall, 25 female and 37 male patients could be analysed whereas one patient’s gender was unknown. Complication of wisdom-tooth infection (*n* = 3) as well as the results of dental procedures like dental extraction (*n* = 7), mandibular nerve block (*n* = 4), periodontitis therapy (*n* = 1) were reported as MOT cases. From the 15 reported cases that appeared after dental treatment like extraction or local anesthesia the medial pterygoid (*n* = 10) was the most affected muscle. Hereof, females were more affected (*n* = 9) than males (*n* = 6). The most reported clinical symptom of MOT was trismus (*n* = 54), followed by swelling (*n* = 17) and pain (*n* = 13). One clinical case provided by the authors was detected.

**Conclusions:**

Dental procedures, such as local anesthesia or extractions, may cause MOT of the masticatory musculature. Demographical analyses demonstrate that females have a higher risk of developing MOT with respect to dental treatment. The most important treatment option is surgical excision. Subsequent physical therapy can have beneficial effects. Nevertheless, a benefit of interpositional materials and drugs as therapy of MOT of the masticatory muscles has not yet been proven. Myositis ossificans progressiva has to be excluded.

**Electronic supplementary material:**

The online version of this article (10.1186/s13005-018-0180-6) contains supplementary material, which is available to authorized users.

## Background

Myositis ossificans describes a heterotopic bone formation within a muscle. Depending on its cause, the syndrome was classified into two different groups: myositis ossificans progressiva (MOP), also known as fibrodyplasia ossificans progressiva which describes a genetic autosomal dominant genetic disease, and myositis ossificans traumatica (MOT). According to its name MOP develops systemically in muscles, ligaments, fascia, and tendons [1]. The prognosis for MOP is generally poor [2, 3]. However, MOT, which is also called myositis ossificans circumscripta, is characterized by ectopic bone formation within muscles and other soft tissues as a result of a preceded trauma [4]. Recent literature also defines further types of myositis ossificans like post-infectous myositis ossificans [5] or idiopathic myositis ossificans [6]. MOT is mostly reported in the orthopedic literature as a result of repeated trauma in muscles like quadriceps femoris. In masticatory muscles, however, MOT is a rare condition which was first reported by Ivy and Eby in 1924 affecting the masseter muscle [7]. In this sense, trismus is the most frequent symptom in the masticatory muscles [8]. The diagnosis MOT can be made if trauma, characteristic clinical and radiological signs, as well as histopathological confirmation are presented [9]. Differential diagnosis must be performed to exclude malignancies like sarcomas, or chondrosarcomas, as well as other neoplasias like osteoma, haemangioma, osteochondroma, or nodular fascitis [10]. Also the anchored disc phenomen and myofibrotic contracture of muscle should be considered [1]. The exact mechanism of the pathogenesis of MOT is unclear. Nevertheless, traumatic, iatrogenic lessions caused by the dentist such as extractions, mandibular block, or periodontal therapy are suspected to be a triggering factor similary to infections like pericoronitis [2, 5, 10–21] . Therefore, the aim of this article was to analyse and interpret the existing literature reporting MOT of masticatory muscles and compare the results with the authors own clinical experience with MOT. The focused question to be answered in this review was: what etiological factors, clinical symptoms, diagnostic imaging and treatments options are reported in current literature to the prevention, diagnosis and therapy of MOT of the masticatory muscles?

## Methods

### Literature review

#### Protocol

The literature search was conducted in accordance to the guidelines available at the “Preferred Reporting Items for Systematic Reviews and Meta-Analyses” (PRISMA) [22].

#### Eligibity criteria

The inclusion criteria consisted of studies describing clinical data reporting on myositis ossificans of the masticatory muscles since the year of the first report (1924) up to date. Due to the lack of clinical trials regarding this issue, no restriction was applied to the study design. Conversely, literature review, books or abstracts or those written in other language than english were excluded from this study.

#### Search strategy

A search strategy was constructed based on PICOS (P = patients; I = Intervention; C = Comparison; O = Outcome, S = Study design), as described in Table 1. The search was conducted in PubMed/Medline database from July to October 2016. Additionally, a manual search was performed based on the references of the screened articles.

**Table 1:** Search strategy constructed based on PICOS

ICOS	Search terms
P = Patients with MOT	• “myositis ossificans traumatica AND masticatory muscle” • “myositis ossificans traumatica AND masseter” • “myositis ossificans traumatica AND pterygoid” • “myositis ossificans traumatica AND temporalis” • “myositis ossificans circumscripta AND masticatory muscle” • “myositis ossificans circumscripta AND masseter” • “myositis ossificans circumscripta AND pterygoid” • “myositis ossificans circumscripta AND temporalis” • “fibrodysplasia ossificans circumscripta AND masticatory muscle” • “fibrodysplasia ossificans circumscripta AND masseter” • “fibrodysplasia ossificans circumscripta AND pterygoid” • “fibrodysplasia ossificans circumscripta AND temporalis”
I = Ossification of masticatory muscles	
C = −	
O = Diagnosis, prevention and treatment	
S = clinical studies, case reports	

#### Study selection

The study selection was independently performed by two reviewers (MH and LH) and, in case of disagreement, a third reviewer (JK) was consulted. First, the articles were screened based on the review of titles and abstracts. Thus, the screened articles were selected for full-text reading and only those considered relevant for this review were included for analysis.

#### Data collection process and items

The first reviewer (MH) extracted the relevant data from the eligible articles and organized them in tables, which were then crosschecked by the second reviewer (LH). The extracted data comprised information regarding gender and age of the affected patient, chief-compliant, affected muscle, history of trauma, treatment protocol, surgical intervention, and follow-up assessment.

#### Risk of bias within studies

The qualitative assessment of the studies was performed using a critical appraisal checklist for case reports [23]. The original check-list consisted of 8 items assessing the quality of case reports. For this study, one item of the original check-list was excluded (“Were adverse events or unanticipated events identified and described?”), as this was not applicable for the most part of the selected studies. All items were marked as yes, no, or unclear. Further, the percentage of positive response (yes) was calculated for each study (Additional file 1).

### Clinical case reported by the authors

The ethical approval for this study was obtained from the ethical review committee (Ref. no. 2017–052-f-N), Ethikkommission der Ärztekammer Westfalen-Lippe und der Westfälischen Wilhelms-Universität, Münster, Germany.

The electronic documentation system, which was maintained in our Dental-Clinic (University Hospital Münster) since 2010, was screened for cases of MOT. The following (german) search terms were used:

- Myositis ossificans
- MOT
- Heterotrope Ossifikation
- Fibrodyplasia ossificans

## Results

### Literature review

#### Study selection

**Fig. 1:**
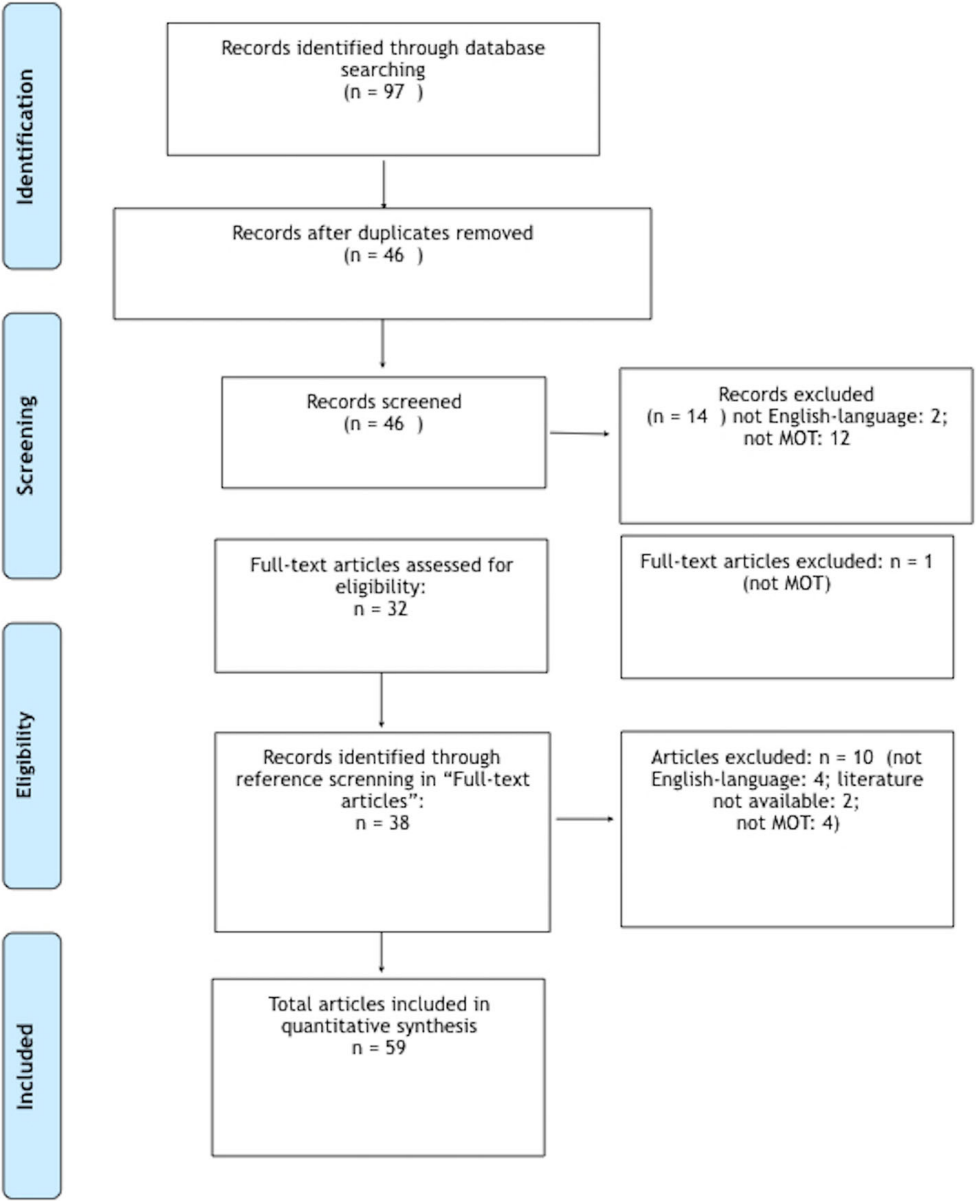
Data analyses of recorded literature for MOT of the masticatory muscles according to PRISMA-Guidelines

A first literature search in PubMed database with the keywords indicated in *Methods* displayed 97 entries. After removing duplicates, 46 articles remained which underwent preselection by screening their abstracts. During the preselection round, two articles were excluded since they were not published in English language (Italian, Turkish) and further 12 articles were eliminated since they did not describe MOT. From these 12 excluded reports, 11 represented MOP cases and one reported about the Carey-Fineman-Ziter syndrome. Subsequently, 32 full-length articles were selected of which one was further excluded because of not detailing MOT. Screening of the references from these selected 31 articles led to further inclusion of 38 articles from which four were rejected again due to publication in national language (German: 2, Japanese: 1, Russian: 1), not describing MOT (*n* = 4), or unavailability (*n* = 2). The mode of literature search was summarized in Fig. 1.

As a final result, it was possible to provide 59 articles reporting about 63 cases of MOT of the masticatory muscles in this study. The study characteristics of the included articles are described in Table 2.

**Table 2:** Review and data summary of MOT of the masticatory muscles cases reported in the literature

Author	Gender, Age	Muscle, side	Chief complaints	History of Trauma	Treatment	Time intervall from trauma to treatment	Further Treatment	Outcome	Follow-up, SKD	Radiology
Fité-Trepat et al. 2016 [10]	Female, 49	Masseter, left side	Trismus, pain, swelling	Repetitive wisdom teeth infection	Excision with 1 cm of tumor-free margins	3 months	None	No recurrence	3 months,	Orthopantomography, CT
Torres et al. 2015 [11]	Female, 36	Medial pterygoid, right side	Trismus, pain, swelling	Extraction upper right wisdom teeth, 4 months later excision of MO alio loco with recurrence	Excision, abdominal fat graft	> 5 months after first surgery	Physical therapy for 1 month	Recurrence	2 months	Orthopantomography, CT, MRI
Mashiko et al. 2015 [31]	Male, 36	Masster bilateral	Trismus, MIO 10 mm	Frequently abused about the face 15 years ago	Osteotomies bilateral, coronoidectomy bilateral	15 years	Physical therapy for 2 months	No recurrence, MIO 36 mm	12 months	CT, PET-CT
Jiang et al. 2015 [5]	Female, 42	Medial and lateral pterygoid right side	Trismus, MIO 2 mm	Wisdom teeth infection	Exzcision, coronoidectomy; pedicled buccal fat pad	36 months	Physical therapy, Celecoxib 200 mg 2xd for 1 week	No recurrence, MIO 25 mm	36 months	Orthopantomography, CT, MRI
Kumar et al. 2014 [32]	Male, 26	Masseter, left side	Painless swelling, MIO 38 mm	Epileptic with multiple falls	Excision	30 months	None	Unknown	Unknown	Orthopantomography, CT, MRI
Almeida et al. 2014 [30]	Female, 12	Lateral pterygoid, left side	Trismus, MIO 10 mm	Unknown	Excision, fat pad	Unknown	Physical therapy, corticosteroids	Recurrence	1 month	CT
Boffano et al. 2014 [26]	Female, 37	Medial pterygoid, left side	Trismus, MIO 5 mm	Trauma: blow of the lef side of her face	Excision together with left coronoid and condyle, TMJ	24 months	Physical therapy	No recurrence, MIO 31 mm	36 months	Orthopantomography, CT
Reddy et al. 2014 [33]	Male, 21	Medial pterygoid and temporalis, left side	Trismus, MIO 15 mm, swelling	Trauma: hit by a heavy vehicle jack rod	First surgery: suspected haematoma eliminated- > MIO 2 mm after 6 weeks. Second surgery: Excision and coronoidectomy	6 weeks	Physical therapy	No recurrence, MIO 30 mm	6 months	CT/MRI
Spinizia et al. 2014 [17]	Male, 30	Lateral pterygoid, left side	Trismus, MIO 10 mm	Trauma: motorcycle ccident	Conservative	1 month	Physical therapy	No recurrence,MIO 30 mm	12 months	CT
Schiff et al. 2013 [29]	Female, 41	Temporalis, left side	Trismus, MIO 2 mm, swelling	Unknown	Excision, coronoidectomy	Unknown	Physical therapy	No recurrence, MIO 51	8 months	Orthopantomography, CT
Jayade et al. 2013 [34]	Female, 25	Lateral and medial pterygoid right side; temporalis left side	Trismus, pain, swelling	Unknown	Excision, coronoidectomy left side	Unknown	Physical therapy	No recurrence, MIO 39 mm	3 months	Orthopantomography, posteroanterior, CT, MRI
Piombino et al. 2013 [35]	Female, 62	Masseter, right side	Trismus	Unknown	Excision	Unknown	None	No recurrence	24 months	Orthopantomography, CT
Nemoto et al. 2012 [36]	Male, 39	Masseter bilateral; lateral pterygoid left side; temporalis left side	Trismus, MIO 5 mm	Trauma: repeatedly struck with a plastic hammer	Excision masseter bilateral, coronoidectomy bilateral	12 months	Physical therapy	No recurrence, MIO 37 mm	12 months	CT, posteroanterior
Choudhary et al. 2012 [37]	Male, 31	Medial pterygoid, left side	Trismus, MIO 8 mm	Trauma: road traffic crash	Excision	24 months	Physical therapy	No recurence, MIO 27 mm	30 months	Orthopantomography, CT, lateral oblique, paranasal view
Guarda-Nardini et al. 2012 [38]	Male, 50	Temporalis, right side	Trismus, MIO 12 mm, pain	Trauma: hited by a piece of furniture	Excision, coronoidectomy	6 months	Physical therapy	No recurrence, MIO 35 mm	6 months	CT, MRI
Reymond et al. 2011 [39]	Male, 22	Masseter, right side	Trismus, MIO 10 mm, swelling	Trauma: assault and battery	Conservative	Unknown	Physical therapy	Remission	6 months	Orthopantomography, CT
Wanyura et al. 2011 [40]	Male, 28	Temporalis, left side	Trismus, MIO 10 mm	Trauma: struck with a fist	At first conservative treatment for 5 months: no succes. First surgery: Excision- > Recurrence. Second surgery 5 months later: Coronoidectomy	5 months	Physical therapy	First surgery: recurrence. Second surgery: no recurrence, MIO 40 mm	6 years	CT, MRI
Thangavelu et al. 2011 [12]	Male, 36	Medial pterygoid, left side	Trismus, MIO 3 mm, pain	Extraction left third molar	Excision and osteotomy at ramus in the area of muscle insertion. Abdominal fat pad	5 months	Physical therapy	No recurence, MIO 28 mm	9 months	Orthopantomography, CT
Godhi et al. 2011 [41]	Male, 21	Lateral pterygoid bilateral, temporalis bilateral	Trismus, MIO 5 mm, swelling	Unknown, swelling 6 years ago	Right side: ostectomy, reconstruction plate; left side: coronoidectomy	6 years	Physical therapy	No recurrence	12 months	CT
Ramieri et al. 2010 [42]	Male, 64	Medial pterygoid, left side	Trismus, MIO 15 mm	Unknown	Excision	Unknown	None	Unknown	Unknown	CT, MRI
Trautmann et al. 2010 [2]	Male, 33	Medial pterygoid, left side	Trismus, MIO 5 mm, swelling	Mandibular block	First surgery: coronoidectomy- > relapse. Second surgery: 3,5 years later: excision- > relapse	First surgery:2 months after mandibular block	None	Recurrence	3 years after second surgery	Orthopantomography, CT, MRI, DVT
Bansal et al. 2009 [13]	Female, 20	Medial pterygoid, right side; (buccinator, right side)	Trismus, MIO 1 mm	Extraction	Excision along with the overlying mucosa, bilateral coronoidectomy	24 months	None	No recurrence	12 months	Orthopantomography, CT
Conner and Duffy 2009 [14]	Female, 18	Medial pterygoid and temporalis right side, afterwards masster and (sternocleidomastoideus) left side	Trismus, MIO 4 mm	Extraction of all 4 third molars	First surgery: excision and coronoidectomy- > recurrence. Second surgery: modified radical neck dissection, excision and resection lingual surface of the mandible, reconstruction plate- > recurrence. Third surgery: disrticulation of right condyle, excision and resection	First surgery after 9 months	Didronel	First surgery: recurrence. Second surgery: recurrence, third surgery: no recurrence, MIO 25 mm	18 months	Orthopantomography, CT, MRI, Scintigraphy
Kruse et al. 2009 [43]	Female, 35	Masseter bilateral	Trismus, MIO 10 mm	Intubated for 4 weeks	Active mouth opening	–	Physical therapy	Unchanged	8 months	Orthopantomography, CT
Rattan et al. 2008 [28]	Male, 45	Medial pterygoid, left side	Trismus, MIO 7 mm	Injection with absolute alcohol in left alveolar nerve	Excision, buccal fat pad	3 years	Physical therapy	No recurrence, MIO 45 mm	24 months	Orthopantomography, CT
Manzano et al. 2007 [44]	Male, 51	Temporalis, right side	Trismus, MIO 13 mm	Trauma 25 years ago	Excision	25 years	Physical therapy	No recurrence, MIO 38 mm	12 months	Orthopantomography, CT
Uematsu et al., 2005 [45]	Female, 38	Temporalis, left side	Pain, swelling	Unknown	Excision	Unknown	None	Unknown	Unknown	CT, MRI
Yano et al. 2005 [46]	Male, 34	Masster bilateral; temporalis left side	Trismus, MIO 5 mm	Trauma: kidnapped and outraged	Excision, coronoidectomy left side	6 months	Physical therapy	No recurrence, MIO 40 mm	10 months	CT, Cephalography
St.-Hilaire et al. 2004 [15]	Male, 68	Masster left side, medial pterygoid left side, temporalis left side	Trismus, MIO 5 mm	Mandibular block	Excision, coronoidectomy	5 weeks	Physical therapy	No recurrence, MIO 40 mm	42 months	Orthopantomography, CT
Aoki et al. 2002 [8]	Male, 44	Masseter left side, medial pterygoid right side	Trismus, MIO 7 mm, pain	Trauma: Blow	Physical therapie for 2 months: no improvment, then surgery with excision masster muscle, 10 days later: recurrence and ossification medial pterygoid right side	12 months	Physical therapy	recurrence	30 months	Orthopantomography, CT, MRI, Scintigraphy
Kim et al. 2002 [16]	Female, 30	Lateral pterygoid bilateral	Trismus, MIO 8 mm	Mandibular block	First surgery: excision, coronoidectomy- > recurence. Second surgery- > recurrence. Third surgery: excision + abdominal fat graft- > recurrence. Fourth surgery	3 years	Radiation therapy, physical therapy, indomethazin, prednisolone, Etidronat	Multiple recurrence, no recurrence after fourth surgery, MIO 22 mm	6 years	Orthopantomography, CT, MRI, Scintigraphy
Saka et al. 2002 [47]	Male, 33	Temporalis, left side	Trismus, pain, swelling	Blunt trauma	Excision	3 weeks	None	No recurrence	4 years	Orthopantomography, CT, MRI, Ultrasound
Mevio et al. 2001 [25]	Female, 55	Temporalis, right side	Trismus, MIO 6 mm	Extraction	Excision, coronoidectomy	18 months	Physical therapy	No recurrence	6 months	CT
Takahashi and Sato 1999 [48]	Female, 71	Medial pterygoid, left side	None	Unknown	Excision	Unknown	None	No recurrence	12 months	Orthopantomography, CT
Spinazze et al. 1998 [17]	Female, 55	Medial and lateral pterygoid left side, temporalis left side	Trismus	Mouth kept open for 3 h during periodontal therapy	First surgery alio loco: coronoidectomy- > recurrence. Second surgery: excision, release of muscular attachments, athrotomyand bony ankylosis, placement of Silastic- > recurrence. Third surgery: gap-athroplasty, wide excision, removement of Silastic	Second surgery: 3 months after first surgery. Third surgery: 3 months after second surgery	Didronel, physical therapy	No recurrence after third surgery, MIO 32 mm	3 months	Orthopantomography, CT, MRI
Myoken et al. 1998 [49]	Male, 55	Masster right side, temporalis bilateral	Trismus, MIO 8 mm	Trauma: zygomatic arch fracture	Excision, bilateral coronoidectomy	1 month	None	No recurrence, MIO 38 mm	12 months	CT
Geist et al. 1998 [50]	Male, 44	Masseter left side	Trismus, MIO 5 mm, pain	Trauma: fracture of the left mandible	Excision	12 months	None	Unknown	Unknown	Orthopantomography, half-axial, CT
Steiner et al. 1997 [51]	Male, 40	Masseter left side	Trismus, MIO 5 mm	Trauma: fracture of the mandible	Excision	12 months	Physical therapy	No recurrence, MIO 30 mm	3 months	Orthopantomography, CT
Steiner et al., 1997 [51]	Female, 15	Masseter left side	Trismus, 8 mm	Shotgun wound to the face 7 years ago	Excision	7 years	Physical therapy	No recurrence, MIO 26 mm	Unknown	CT
Tong et al. 1994 [52]	Female, 73	Medial pterygoid bilateral	None	Unknown	None	Unknown	None	Unknown	Unknown	CT
El-Labban et al. 1993 [53]	Male, 42	Masster, side unknown	Trismus	Trauma: blow to the side 6 months before	Unknown	Unknown	Unknown	Unknown	Unknown	Unknown
Parkash and Goyal 1992 [18]	Male, 28	Medial pterygoid, left side	Trismus, MIO 0 mm	Pericoronitis left third molar	First surgery: condylectomy and coronoidectomy- > recurrence Second surgey: excision	6 ½ years	Physical therapy	Recurrence, after second surgery: MIO 20 mm	3 months	Orthopantomography, CT
Nilner and Andersson 1989 [54]	Male, 57	Medial pterygoid, right side	Trismus	Injection with alcohol in right alveolar nerve	None	–	None	Unknown	8 years	Orthopantomography, CT, TMJ radiograph
Lello and Makek 1986 [19]	Female, 31	Masster left side	Trismus, MIO 10 mm, pain, swelling	Mandibular block	Excision	5 weeks	None	No recurrence, MIO 40 mm	4 years	Orthopantomography, posteroanterior Scintigraphy
Lello and Makek 1986 [19]	Male, 32	Masster, left side	Trismus, MIO 10 mm, swelling	Trauma: blow to the left mandible	Excision	2 months	None	No recurrence	5 years	Unknown
Lello and Makek 1986 [19]	Male, 34	Temporalis left side	None	Trauma: motor vehicle accident	Excision	9 months	None	No recurrence	4 years	CT
Wiesenfeld et al. 1985 [55]	Female, 10	Temporalis right side	Painless swelling	Unknown	Excision	Unknown	None	No recurrence	6 months	Orthopantomography, CT
Arima et al. 1984 [56]	Male, 25	Masseter, left	Trismus, MIO 11 mm, pain	Trauma: contusion in a fight	Excision	6 months	None	No recurrence, MIO 47 mm	11 months	Posterioanterior
Abdin and Prabhu 1984 [57]	Female, 43	Lateral pterygoid left side	Total trismus, painless swelling	Huge painful swelling at the age of 19	Excision	24 years	Physical therapy	No recurrence, MIO 30 mm	6 months	Orthopantomography
Christmas and Ferguson 1982 [58]	Male, 51	Masseter, left side	Trismus, MIO 10 mm, swelling	Trauma: falling from horse and striking against a fence post	Excision	18 months	None	No recurrence, MIO 40 mm	4 months	Posterioanterior
Plezia et al. 1977 [59]	Female, 47	Masseter, left side	Trismus, MIO 8 mm	Trauma: blow	Excision	2 months	None	No recurrence, MIO 44 mm	unknown	Posterioanterior
Narang and Dixon 1974 [20]	Male, 50	Medial pterygoid, right side	Trismus, MIO 12 mm	Extraction	First surgery: excision- > recurrence Second surgery: excision, coronoidectomy, insetion of silastic	First surgery:15 months Second surgery: 1 month	Physical therapy	No recurrence, MIO 49 mm	unknown	Cephalography, posterioanterior
Hatzifotiadis 1970 [60]	Male, 50	Masseter, left side	Trismus, MIO 5 mm, swelling	Trauma: fallen on iron peg	First: conservative treatment without succes for 2 months. Surgery: Excision	4 months	Physical therapy, acrylic appliance for 2 days	No recurrence	12 months	Radiograph
Trester et al. 1969 [61]	Female, 29	Masseter, left side	Trismus, MIO 3–4 mm, swelling	Trauma: epileptic seizure- > blow	Excision- > recurrence, than physical therapy	1 month	Physical therapy	Recurrence after surgery- > with physical therapy: MIO 25 mm	3 months	Posterioanterior
Vernale 1968 [62]	Male, 31	Masseter, right side	Trismus, pain, swelling	Trauma: car accident	Excision	1 month	None	No recurrence	2 months	Posterioanterior
Vernale 1968 [62]	Male, 29	Masseter, left side	Trismus, MIO 4 mm	Trauma: blow	Excision	4 months	None	No recurrence	6 years	Posterioanterior, right and left lateral oblique
Shawkat 1967 [21]	Male, 24	Masseter, temporalis, (mylohyoid), left side	Facial paralysis	Extraction left maxillary molar region	Unknown	Unknown	None	Unknown	Unknown	Cephalography
Parnes and Hinds 1965 [63]	Female, 27	Masster, left side	Trismus, MIO 10 mm, pain	Trauma: beaten with a fist	Excision	1 month	None	No recurrence, MIO 25 mm	Unknown	Posterioanterior, right and left lateral oblique
Hellinger 1965 [64]	Female, 21	Masster, temporalis, (buccinator) pterygoid, left side	Trismus, MIO 3–4 mm	Unknown	Excision	12 years	None	No recurrence	6 months	Posterioanterior, lateral oblique
Goodsell 1962 [65]	Male, 39	Masseter, right side	Trismus, pain, swelling	Trauma: blow	Excision	5 weeks	None	No recurrence	Unknown	Unknown
Kostrubala and Tailbot 1948 [66]	Male, 21	Masseter, right side	Trismus	Trauma: struck by an enemy bullet	First surgery:Excision- > recurrence Second surgery: excision + dermal graft	6 months, second surgery after 4 months	Before surgery: physical therapy- > no succes	After second surgery: no recurrence	9 months	Laminograph
Nizel and Prigge 1946 [4]	Male, 21	Masseter, right side	Trismus, MIO 4 mm	Trauma: perforating wound	Conservative treatment	4 months	Counter-trismus appliance	MIO 21 mm		Posterioanterior
Ivy and Eby 1924 [7]	Unknown	Masseter, left side	Trismus	Trauma: wounded by a small shell fragment	Excision	Unknown	Trismus apparatus	Full extent of opening achieved immediaely postoperative	Unknown	Radiograph

### Results of individual studies

#### Gender prevalence and age

Overall, 63 patients were reported involving 25 female and 37 male patients that were analysed. One patient’s gender was not indicated. Therefore, approximately two out of three patients were male. The age ranged from 10 to 73 years in the female group (mean: 38.6 years). In the male group the age ranged from 21 to 68 years (mean: 37.4 years).

#### Affected muscle

The most frequent affected muscle was the masseter muscle, which was hit 35 times (left side: 23-fold, right side: 11-fold, side unknown: 1-fold). The temporalis muscle was concerned 22 times (left side: 14-fold, right side: 8-fold) followed by the medial pterygoid muscle with 21 cases (left side: 12-fold, right side: 9-fold). The lateral pterygoid muscle was affected 12 times (left side: 8-fold, right side: 4). In18 cases more than a single muscle was hit by MOT.

#### Clinical symptoms

The most reported clinical symptoms of MOT were trismus (*n* = 54), followed by swelling (*n* = 17), and pain (*n* = 13). Facial paralysis was outlined in one case, while three cases were reported to be devoid of any clinical symptoms. Trismus ranged from 0 to 15 mm (mean: 7.3 mm).

#### Kind of trauma

As triggering event, strokes or falls were reported most frequently (*n* = 21), while in 12 cases a triggering event was unknown. Car accidents seemed to be the reason for five cases of MOT but MOT development due to dental procedures like dental extraction (*n* = 7), mandibular nerve block (*n* = 4), periodontitis therapy (*n* = 1), or as a result of alcohol injection into the alveolar nerve (n = 2) were also described. MOT as a complication of wisdom-tooth infection was reported in three cases. Furthermore, occurrence of MOT was published as a consequence of post-fracture (*n* = 3), gunshot injury (n = 2), perforating wound (*n* = 1), injury caused by a shell (n = 1), and after intubating a patient for 4 weeks (n = 1).

#### Time interval from trauma to treatment

Time intervals from trauma to treatment were not addressed in 13 cases, while in two reports no treatment was initiated. In 48 cases, time intervals were reported, which ranged from 3 weeks to 25 years, whith an average time of 31 months.

#### Treatment

The most frequent described treatment for MOT was surgical excision (*n* = 23) followed by surgery and physical therapy (*n* = 22). In addition to surgery, interposition grafts and physical therapy were performed by five authors, interponate with silastic and physical therapy was reported in one case, while another author described interponate with silastic, physical therapy, and drug administration using diodronel. Didronel was administered in addition to surgery according to one report. The use of dermalgraft in combination with surgical excision was also reported in one case. The use of radiation and surgery in combination with physical therapy and drug administration with indomethacine and etidronate was furthermore published in one case. Exclusive physical therapy was done in four cases, while treatment in two reports was not indicated. Multiple surgeries were necessary in 9 patients. Two patients were not treated at all.

#### Clinical outcome: No recurrence

In 41 cases, no recurrence was reported after the first surgery. Nineteen out of these 41 cases were treated with a combination of surgery and physical therapy while 20 of 41 cases underwent exclusively surgery. One patient was treated with surgery in combination with physical and pharmacological therapy, while another patient was handled with surgery in combination with interponate and physical therapy. In contrast, recurrence took place in 11 cases whereas no treatment was performed or the outcome was not outlined in 11 cases.

#### Clinical outcome: Recurrence

Recurrence was reported in a total of 11 cases. In 7 out of these cases multiple surgeries were performed which stopped any further recurrence. In four reports, unsuccessful treatment of MOT hampered recurrence analysis.

#### Clinical outcome: Recurrence in correlation with time of treatment

To evaluate the clinical outcome “recurrence” in correlation with time of treatment, two groups were defined. In the first group, surgery was performed less than 6 months after trauma (*n* = 21). In this group five cases with recurrence were stated. In the second group, the interval from trauma to treatment was longer than 6 months (*n* = 27). In that herein also five cases with clinical recurrence occurred. In one case undergoing recurrence no interval from trauma to treatment was indicated. In a total of 13 reports, the interval from trauma to treatment was not noted and in two cases no treatment was initiated.

#### Clinical outcome: Recurrence in correlation with the type of treatment

Recurrence after the first treatment was found in 3 cases in which only surgery took place. Surgery in combination with physical therapy led to 3 cases of recurrence. Surgery in combination with fat pad and physical therapy led to recurrence in two reports, while recurrence also occurred to a patient who was treated with surgery in combination with diodronel. Recurrence was also reported during treatment with surgery in combination with radiation, indomethacine, diodronel, and physical therapy, as well as surgery with silastic interponate, diodronel, and physical therapy.

#### Clinical outcome: Maximal incisal opening (MIO) development

In the group of successful treated patients, 20 authors reported about the development of MIO before and after therapy. MIO ranged from 15 to 49 mm in length with a mean of 29.6 mm. Only physical therapy (*n* = 1) yielded a 20 mm long MIO. Surgery in combination with fat pad (*n* = 3) resulted in a MIO of 28.6 mm length (range: 23 mm–38 mm), while the MIO of patients with surgery in combination with physical therapy (*n* = 12) exhibited a MIO of 27.2 mm length (range: 15 mm–49 mm). Surgery alone (*n* = 4) yielded a MIO of 31.3 mm length (range: 30 mm–35 mm).

#### Risk of bias within studies

In general, the risk of bias was considered low, since most part of the case reports were described in accordance to the check-list. Only 2 studies showed a percentage of positive response lower than 60% (Supplement 1).

### Clinical documentation system screening

After searching the clinical documentation system of the University Hospital Münster only one self-generated entry for MOT could be recovered.

### Clinical case reported by the authors

**Fig. 2:**
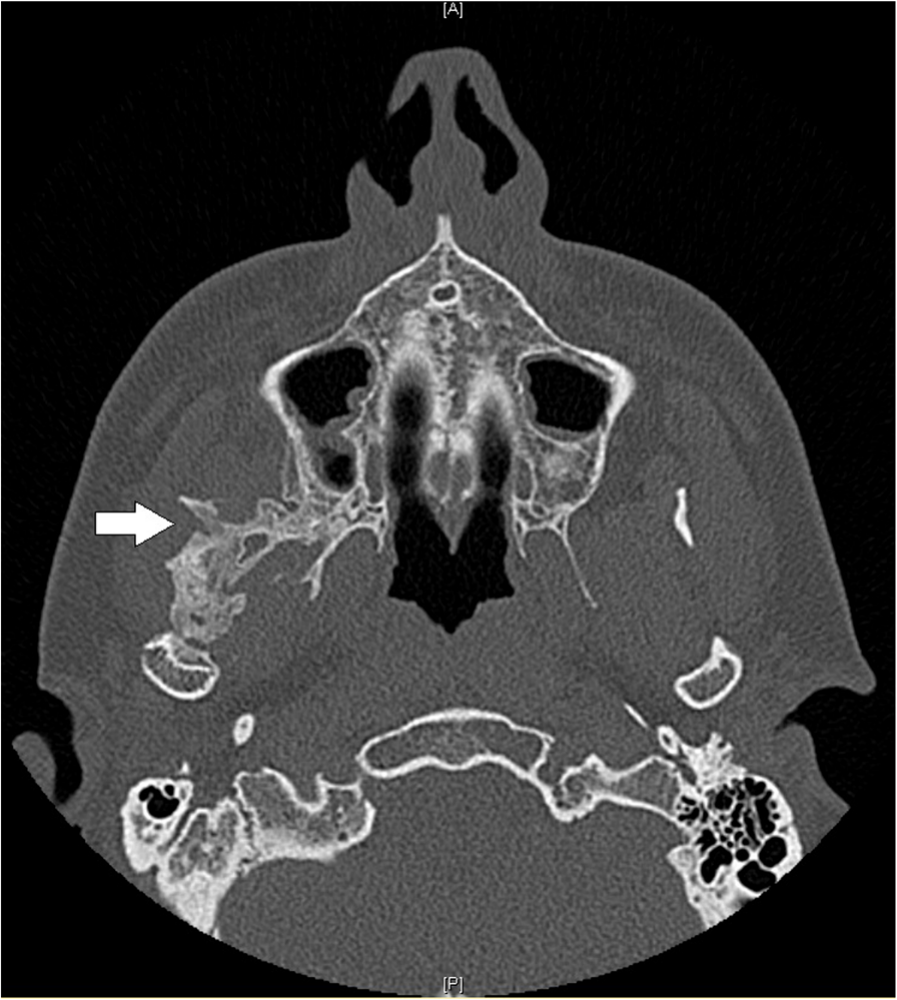
Cone beam scan showing calcification of the right medial pterygoid

**Fig. 3:**
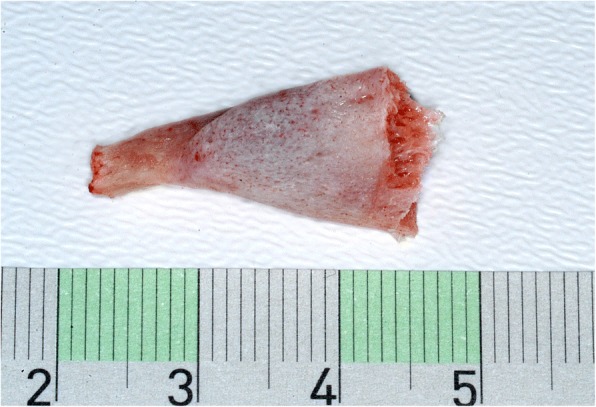
Piece of the excised solid bone mass

**Fig. 4:**
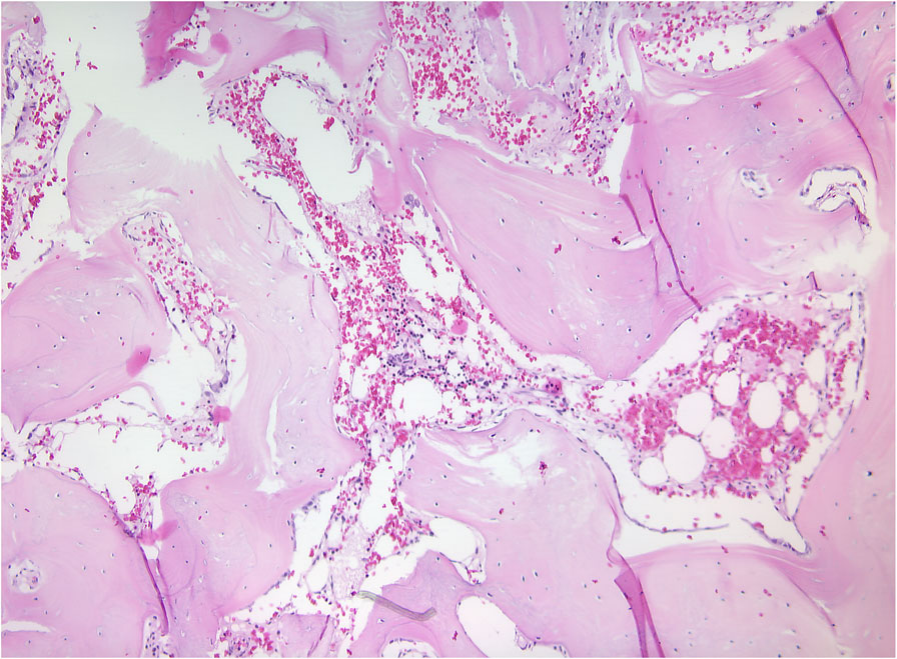
Microscopic image of lesion demonstrating sclerotic, solid and cancellous bone with fatty bone marrow. (HE, magnification: 10-fold)

**Fig. 5:**
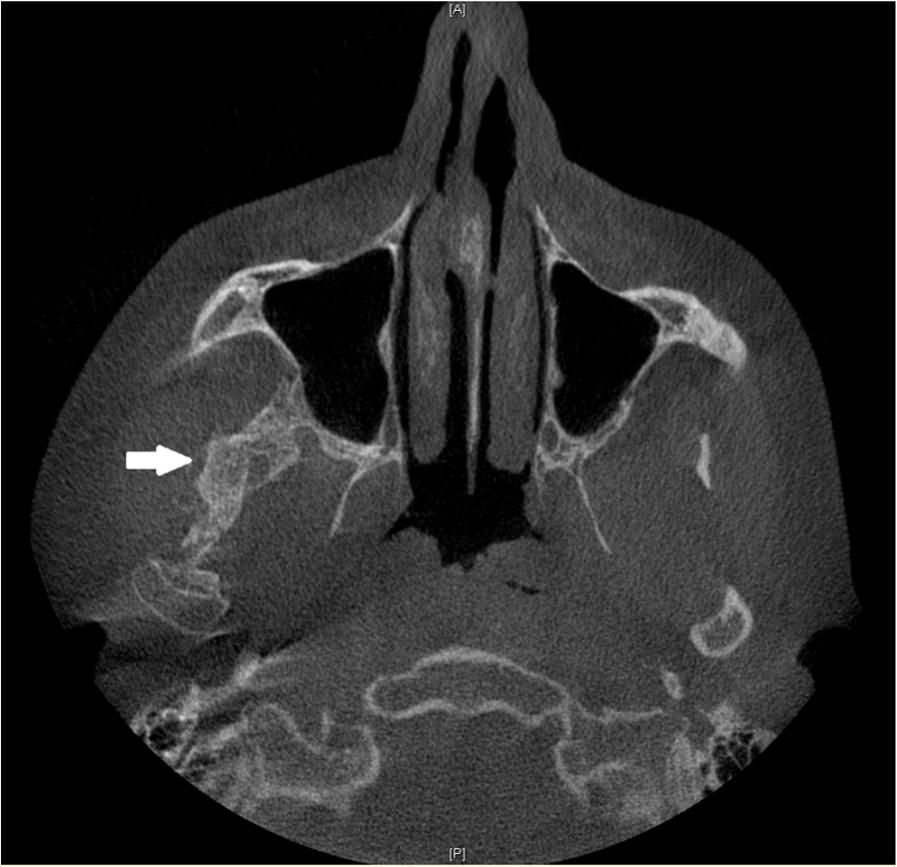
Digital volume tomography showing recurrence of calcification

A 28 year-old male was referred to our Clinic of Cranio-Maxillofacial Surgery with trismus in March 2016. The patient was not able to open or to close his mouth and, moreover, he was unable to protrude or to produce a lateral excursion. So he possessed an interincisal mouth opening of 5 mm. The patient indicated that he underwent a filling therapy on the right mandible molar by his dentist 7 months ago. As according therapy a right mandibular nerve block was performed. Four weeks later the patient developed trismus. His dentist described oral antibiosis and physical examination. However, no clinical improvement was observed. Therefore, the patient was referred to a Clinic of Cranio-Maxillofacial Surgery where the diagnosis of pericoronitis of the lower right third molar was stated. Extraction of the right upper and lower third molar and a forced mouth-opening was performed under general anesthesia. Subsequently, the trismus disappeared but reappeared 2 weeks later. Because of this relapse, coronoidectomy was performed on the right side. Consequently, the trismus disappeared, but a relapse reoccurred a few weeks later. A multislice computer tomography (CT) of the head was performed and the CT revealed a calcification of the right medial pterygoid muscle (Fig. 2). Due to the given diagnosis of MOT of the right medial pterygoid, the patient was finally referred to the Clinic of Cranio-Maxillofacial Surgery at the University of Münster. For excluding MOP, we referred the patient to the department of human genetics. Indeed, MOP could be excluded and also all laboratory test results ranged within normal limits, including the resulting values for calcium, phosphate, alkaline phosphatase and parathyroid hormone measurements. Thus, we decided to perform renewed surgery 6 months after the last surgical intervention. Pre-operative radiation was performed with 6 Gy as single-dose radiation. Surgical excision of the ossified right medial pterygoid muscle was performed through combined intra- and extraoral access under general anesthesia. During this intervention, solid bone mass could be excised (Fig. 3). Histopathological analysis confirmed the diagnosis of MOT (Fig. 4). Physical therapy was started 2 days after surgery and 1 week after surgical intervention the patient could be released. Post-operative long-term application of ibuprofen 400 mg was performed for 2 weeks. At this time point, the MIO reached 23 mm in length. The patient was instructed to perform intensive physical therapy with an functional orthodontic gadget, the so-called “Jeckel-spreader”, for exercising mouth opening. This device serves for mobilisation of the masticatory muscles. Two weeks later, the MIO still yielded 25 mm in length. Thereafter, the patient stopped physical therapy using the “Jeckel-spreader” against our recommendation. Consequently, the MIO decreased to 10 mm in length. Thus, we advised the patient strongly to restart physical therapy but he declined. Digital volume tomography (DVT) was performed which revealed renewed calcification (Fig. 5). Six months after surgery, MIO exhibited a length of about 8 mm. This enabled the patient to eat, to perform and to do a small lateral excursion.

**Fig. 6:**
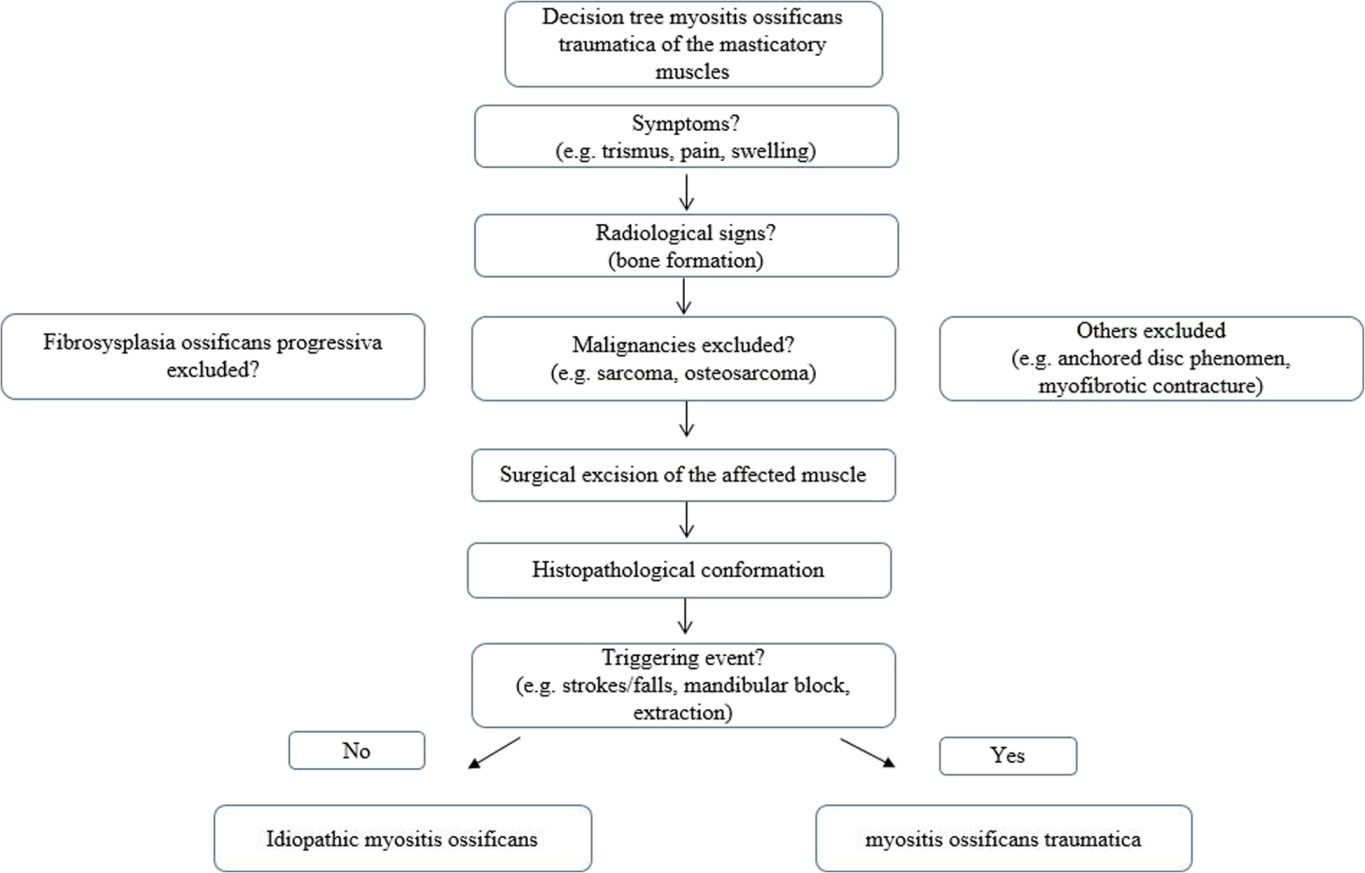
Decision tree for diagnosis and treatement of myositis ossificans traumatica

We have derived a decision tree for diagnosis and treatement of MOT (Fig. 6).

#### Discussion

The pathogenesis of MOT has not been finally clarified. In 1924, Carey [24] already listed four major theories for the development of MOT: 1) Displacement of bony fragments into soft tissue and hematoma with subsequent proliferation; 2) detachment of periosteal fragments into surrounding tissue with proliferation of osteoprogenitor cells; 3) migration of subperiostal osteoprogenitor cells into surrounding soft tissue through periosteal perforations induced by trauma; 4) differentiation of extraosseous cells exposed to bone morphogenic proteins. The results of the present study confirm the assumption,that multiple processes lead to the development of MOT. If a triggering event is present at all, its nature seems to be too heterogenous from case to case to support the theory of a single initiating cause. In 12 of the cases summarized here, no specific triggering traumatic event was identified (idiopathic myositis ossificans). Nevertheless, it seems that minor traumatic lesions unnoticed by these patients could be a possible cause. According to Torres [11] the intensity of the trauma may not be related to the occurrence of MOT. This statement could explain why no cases of MOT occurring in individuals that pursue the sport of boxing have been reported in the literature so far. These cases would be expected because of regularly occurring blows to the face and masticatory muscles (especially the masseter and temporal muscles) of boxers. On the other hand, a relation between dental surgery and the onset of MOT seems obvious. There are 7 case reports of MOT with previous tooth extraction [11–14, 20, 21, 25] though it is not possible to fully differentiate whether the extraction or the dental anesthesia in the context with the extraction represents the triggering event. The latter as a cause of MOT was reported in four cases [2, 15, 16, 19]. Mandibular block as reported by Trautmann [2] as well as in our reported case, could be a more possible triggering factor for MOT. Therefore local anesthesia cannot be excluded as a cause of MOT occurring after periodontal treatment, either [17]. Furthermore, three cases of MOT following repetitive wisdom tooth infection have been published [5, 10, 18]. This would represent an additional indication requiring surgical removal of wisdom teeth if normal placement in the row of teeth is not expected.

Trismus is the most frequently observed symptom of MOT in the masticatory muscles which was also presented in our case. In this respect, MOT should be considered in the differential diagnosis in case of persisting trismus without a clinically manifesting cause. In such cases, radiographic findings are being expected only 3–6 weeks after the appearance of clinical symptoms [2].

So far, male patients have been considered as the main group at risk of developing MOT of the masticatory muscles with a male/female ratio of 2.4/1 [26]. However, our data analysis demonstrated a gender-specific difference to a lesser extent with a male/female ratio of approximately 1.5/1. Since however MOT has been frequently related to traumas (e. g. fracture, blow) a possible explanation could be: males might have experienced traumas more often than females and thus also suffer more often from MOT. Of particular interest is the view at the cases of MOT occurring after dental treatment where more women (*n* = 9) were concerned than men (*n* = 6). This means prevalence for female patients of MOT of the masticatory musculature in context of dental treatment with a 1.5/1 ratio.

In most cases of MOT of the masticatory muscles the masseter muscle is the most affected one [10]. However, this is not true for those cases of MOT occurring after dental treatment. Of these cases (*n* = 10), 66% involved the medial pterygoid muscle. Given the potential risk of damaging this muscle in the context of a mandibular nerve block, local dental anesthesia seems to be the cause of MOT here, as potentially in our case. Whether the patient has to be informed about this extremely rare complication remains questionable in view of the large numbers of local dental anesthesia administered daily. On the other hand the consequences represent a severe impairment for the patient. Nevertheless, MOT should be considered in the differential diagnosis in cases of therapy-resistant trismus developing in the weeks after local anesthesia.

Generally, excision of the affected muscle is recommended as treatment of choice [10]. However, there are different opinions about the time when the excision has to be done and about possible additional measures, such as the use of interpositional materials, treatment with drugs, or physical therapy. Some authors recommended [12, 14, 27] that the excision as well as the use of interpositional material should be performed after complete maturation, about 6 to 12 months after initial symptoms. In contrast, other authors prefered excision at an early stage [11]. There were five relapses, both, in the group of early excision (treatment less than six months after first symptoms), and in the group of excision at a later stage (treatment more than six months after first symptoms). However, the group with intervention at a later time point included 27 cases that was somewhat bigger than the early-intervention group (*n* = 21). Nonetheless, it is not possible to make any clear recommendation for the ideal time point of surgical intervention based on these data.

While some authors suggested aggressive physical therapy after surgical excision [17], others advised against this procedure [14]. They feared that physical therapy stimulates bone formation with the consequence of exacerbation of MOT. Of the 22 reported cases undergoing excision combined with physical therapy, 3 cases relapsed. In the group of 23 patients who only underwent excision without physical therapy there were also 3 relapses. In consequence, no difference in the rate of recurrence was found depending on physical therapy.

In addition to excision, − with or without physical therapy, the use of interpositional materials [12, 16, 17, 20, 28] or pharmaceuticals, such as etidronate or ibuprofen [29] have been proposed. Often, these additional measures were applied in clinical cases with multiple recurrences [11, 14, 16, 17, 30] so that the benefit of additional treatment cannot be assessed conclusively.

The major limitation of this review is the rarity of the evaluated condition, resulting in a lack of research sources which could offer reliable evidence-based information. With this regard, all studies selected for this review were case reports, which hampered a deeper analysis of risk of bias of each study. Nonetheless, the present study aimed to offer a guide decision for the management and diagnosis of MOT. Additionally, the case reported described the authors clinical experience regarding this condition and shows a treatment option for patients with MOT.

## Conclusions

Dental procedures, such as local anesthesia or extractions, may cause MOT of the masticatory musculature. Women have a higher risk of developing MOT with respect to dental treatment. The most important treatment option for MOT is surgical excision and subsequent physical therapy can have beneficial effects. A benefit of interpositional materials and drugs as therapy of MOT of the masticatory muscles has not yet been proven. MOP has to be excluded.

## Additional file

Additional file 1. Quality assessment of the included literature. (DOC 139 kb)

## Abbreviations

CT: Computer tomography
DVT: Digital volume tomography
MIO: Maximal incisal opening
MOP: Myositis ossificans progressiva
MOT: Myositis ossificans traumatica
