# Supplementary material for: Myositis ossificans traumatica of the masticatory muscles: etiology, diagnosis and treatment
Source: Head Face Med. 2018 Oct 29;14:23. doi: 10.1186/s13005-018-0180-6 (PMC6206835; doi:10.1186/s13005-018-0180-6)
Supplement: Supplementary file 1 — Quality assessment of the included literature. (DOC 139 kb) [file 13005_2018_180_MOESM1_ESM.doc]

| **Author** | **Were patient’s demographic characteristics clearly described?** | **Was the patient’s history clearly described and presented as a timeline?** | **Was the current clinical condition of the patient on presentation clearly described?** | **Were diagnostic tests or methods and the results clearly described?** | **Was the intervention(s) or treatment procedure(s) clearly described?** | **Was the post-intervention clinical condition clearly described?** | **Does the case report provide takeaway lessons?** | **Total (%)** |
| --- | --- | --- | --- | --- | --- | --- | --- | --- |
| Fité-Trepat et al 2016 ^10^ | Yes | Yes | Yes | Yes | Yes | Yes | Yes | 100 |
| Torres et al. 2015 ^12^ | Yes | Yes | Yes | Yes | Yes | Yes | Yes | 100 |
| Mashiko et al. 2015 ^31^ | Yes | Yes | Yes | Yes | Yes | Yes | Yes | 100 |
| Jiang et al. 2015 ^5^ | Yes | Yes | Yes | Yes | Yes | Yes | Yes | 100 |
| Kumar et al. 2014 ^33^ | Yes | Yes | Yes | Yes | Yes | No | Yes | 85.71 |
| Almeida et al. 2014 ^31^ | Yes | Yes | Yes | Yes | Yes | Yes | Yes | 100 |
| Boffano et al. 2014 ^27^ | Yes | Yes | Yes | Yes | Yes | Yes | Yes | 100 |
| Reddy et al. 2014 ^34^ | Yes | Yes | Yes | Yes | Yes | Yes | Yes | 100 |
| Spinizia et al. 2014 ^18^ | Yes | Yes | Yes | Yes | Yes | Yes | Yes | 100 |
| Schiff et al. 2013 ^30^ | Yes | Yes | Yes | Yes | Yes | Yes | Yes | 100 |
| Jayade et al. 2013 ^35^ | Yes | Yes | Yes | Yes | Yes | Yes | Yes | 100 |
| Piombino et al. 2013 ^36^ | Yes | Yes | Yes | Yes | Yes | Yes | Yes | 100 |
| Nemoto et al. 2012 ^37^ | Yes | Yes | Yes | Yes | Yes | Yes | Yes | 100 |
| Choudhary et al. 2012 ^38^ | Yes | Yes | Yes | Yes | Yes | Yes | Yes | 100 |
| Guarda-Nardini et al. 2012 ^39^ | Yes | Yes | Yes | Yes | Yes | Yes | Yes | 100 |
| Reymond et al. 2011 ^40^ | Yes | Yes | Yes | Yes | Yes | Yes | Yes | 100 |
| Wanyura et al. 2011 ^41^ | Yes | Yes | Yes | Yes | Yes | Yes | Yes | 100 |
| Thangavelu et al. 2011 ^13^ | Yes | Yes | Yes | Yes | Yes | Yes | Yes | 100 |
| Godhi et al. 2011 ^42^ | Yes | Yes | Yes | Yes | Yes | Yes | Yes | 100 |
| Ramieri et al. 2010 ^43^ | Yes | Yes | Yes | Yes | Yes | Unclear | Yes | 85.71 |
| Trautmann et al. 2010 ^2^ | Yes | Yes | Yes | Yes | Yes | Yes | Yes | 100 |
| Bansal et al. 2009 ^14^ | Yes | Yes | Yes | Yes | Yes | Yes | Yes | 100 |
| Conner and Duffy 2009 ^15^ | Yes | Yes | Yes | Yes | Yes | Yes | Yes | 100 |
| Kruse et al. 2009 ^44^ | Yes | Yes | Yes | Yes | Yes | N/A | Yes | 85.71 |
| Rattan et al. 2008 ^29^ | Yes | Yes | Yes | Yes | Yes | Yes | Yes | 100 |
| Manzano et al. 2007 ^45^ | Yes | Yes | Yes | Yes | Yes | Yes | Yes | 100 |
| Uematsu et al., 2005 ^46^ | Yes | Yes | Yes | Yes | No | No | No | 57.14 |
| Yano et al. 2005 ^47^ | Yes | Yes | Yes | Yes | Yes | Yes | Yes | 100 |
| St.-Hilaire et al. 2004 ^16^ | Yes | Yes | Yes | Yes | Yes | Yes | Yes | 100 |
| Aoki et al. 2002 ^8^ | Yes | Yes | Yes | Yes | Yes | Yes | Yes | 100 |
| Kim et al. 2002 ^17^ | Yes | Yes | Yes | Yes | Yes | Yes | Yes | 100 |
| Saka et al. 2002 ^48^ | Yes | Yes | Yes | Yes | Yes | Yes | Yes | 100 |
| Mevio et al. 2001 ^25^ | Yes | Yes | Yes | Yes | Yes | Yes | Yes | 100 |
| Takahashi and Sato 1999 ^49^ | Yes | Yes | Yes | Yes | Yes | No | Yes | 85.71 |
| Spinazze et al. 1998 ^18^ | Yes | Yes | Yes | Yes | Yes | Yes | Yes | 100 |
| Myoken et al. 1998 ^50^ | Yes | Yes | Yes | Yes | Yes | No | No | 71.42 |
| Geist et al. 1998 ^51^ | Yes | Yes | Yes | Yes | Yes | No | Yes | 85.71 |
| Steiner et al. 1997 ^52^ | Yes | Yes | Yes | Yes | Yes | Yes | Yes | 100 |
| Steiner et al, 1997 ^52^ | Yes | Yes | Yes | Yes | Yes | Yes | Yes | 100 |
| Tong et al. 1994 ^53^ | Yes | No | Yes | Yes | No | No | Yes | 71.42 |
| El-Labban et al. 1993 ^54^ | Yes | Yes | No | No | No | No | Yes | 42.85 |
| Parkash and Goyal 1992 ^19^ | Yes | Yes | Yes | Yes | Yes | Yes | Yes | 100 |
| Nilner and Andersson 1989 ^55^ | Yes | Yes | Yes | Yes | Yes | Yes | Yes | 100 |
| Lello and Makek 1986 ^20^ | Yes | Yes | Yes | Yes | Yes | Yes | Yes | 100 |
| Wiesenfeld et al. 1985 ^56^ | Yes | Yes | Yes | Yes | Yes | Yes | Yes | 100 |
| Arima et al. 1984 ^57^ | Yes | Yes | Yes | Yes | Yes | Yes | Yes | 100 |
| Abdin and Prabhu 1984 ^58^ | Yes | Yes | Yes | Yes | Yes | Yes | Yes | 100 |
| Christmas and Ferguson 1982 ^59^ | Yes | Yes | Yes | Yes | Yes | Yes | Yes | 100 |
| Plezia et al. 1977 ^60^ | Yes | Yes | Yes | Yes | Yes | Yes | Yes | 100 |
| Narang and Dixon 1974 ^21^ | Yes | Yes | Yes | Yes | Yes | Yes | Yes | 100 |
| Hatzifotiadis 1970 ^61^ | Yes | Yes | Yes | Yes | Yes | Yes | Yes | 100 |
| Trester et al. 1969 ^62^ | Yes | Yes | Yes | Yes | Yes | Yes | Yes | 100 |
| Vernale 1968 ^63^ | Yes | Yes | Yes | Yes | Yes | Yes | Yes | 100 |
| Shawkat 1967 ^22^ | Yes | Yes | Yes | Yes | No | No | Yes | 71.42 |
| Parnes and Hinds 1965 ^64^ | Yes | Yes | Yes | Yes | Yes | Yes | Yes | 100 |
| Hellinger 1965 ^65^ | Yes | Yes | Yes | Yes | Yes | Yes | Yes | 100 |
| Goodsell 1962 ^66^ | Yes | Yes | Yes | Yes | Yes | Yes | Yes | 100 |
| Kostrubala and Tailbot 1948 ^67^ | Yes | Yes | Yes | Yes | Yes | Yes | Yes | 100 |
| Nizel and Prigge 1946 ^4^ | Yes | Yes | Yes | Yes | Yes | Yes | Yes | 100 |
| Ivy and Eby 1924 ^7^ | No | Yes | Yes | Yes | Yes | No | Yes | 71.42 |
